# Standard operating procedure for optimal identification of mycobacteria using 16S rRNA gene sequences

**DOI:** 10.4056/sigs.932152

**Published:** 2010-09-28

**Authors:** Enrico Tortoli

**Affiliations:** Regional Reference Center for Mycobacteria, Careggi University Hospital, 50134 Florence, Italy

**Keywords:** Mycobacterium, identification, genetic sequencing, 16S rRNA, INSDC partners, genetic database

## Summary

Genetic sequencing is increasingly used for the identification of bacteria and for many of them, including mycobacteria, the analysis of the 16S rRNA gene represents the gold standard. Sequencing technology has greatly improved in recent years and now allows  one  to obtain good quality electropherograms also in non specialized settings. The interpretation of the electropherograms still lacks however precise rules and the achievement of an incorrect identification from an excellent sequence is very frequent, in particular for organism like mycobacteria which are very closely related to each other at the genetic level. Without claiming to be authoritative or comprehensive, this note aims to provide a few simple directions suitable to steer the choice of the best possible identification.

## Introduction

The accurate identification of bacteria is becoming more and more problematic, mainly because of the steady increase of the number of species. The problem is even more complex for what concerns the members of the genus *Mycobacterium* which are genetically more closely related to each other in comparison to microorganisms belonging to other genera [1].

The phenotypic approach based on biochemical and cultural investigations accompanied for many years the glorious history of the International Working Group of Mycobacterial Taxonomy [2-5]. The use of such methods for identification purposes is currently inconceivable. The almost 150 species presently recognized would need the investigation of a huge number of features even in the unrealistic hypothesis they were 100% discriminative and error free.

In mycobacteriology, the switch from the phenotypic approach to the genotypic one has not been as quick as in other microbiological fields. A large popularity was in fact gained in the 1990s by the chemotaxonomic methods based on the analysis of cell wall lipids. Their discriminative power was quite low when using thin layer chromatography [6], but it was considerably better with gas-liquid chromatography [7] or the high performance liquid chromatography [8,9]. In the last years, however, even the most advanced chromatographic methods failed to discriminate within important groups of mycobacteria, in particular the rapid growers, the so called *Mycobacterium avium*-*intracellulare-scrofulaceum* (MAIS) complex and the group of species related to *Mycobacterium simiae* [1].

The Copernican revolution following the extraordinary progress of genetic knowledge produced, for what concerns the identification of mycobacteria, two techniques which still remain very popular worldwide, DNA probe hybridizations and PCR-restriction analysis (PRA). The first, despite the important improvement brought by the implementation of the line-probe technology, is however hampered by the limited number of species it can identify and by several cross-reactions [10]. Suboptimal sensitivity and specificity represent instead the Achilles’ heel of PRA [1].

The sequencing of conserved genetic regions is universally recognized as the gold standard for the identification of mycobacteria [11]. Such technique, limited at first to highly specialized laboratories, has become in recent years very popular mainly due to the robustness of the chemistry (based on the Big Dye Terminator technology) and to the excellent performance of modern automatic instrumentations.

A number of targets useful for identification purpose have been detected within the genome of mycobacteria. The 16S rRNA gene, in particular the trait including the first 500 bp, as the determination of the full gene is needed in a limited number of cases, is universally considered the first choice. The best alternative to 16S rRNA appears to be the hypervariable 400 bp fragment of the *hsp65* gene [12]. Other, more variable, regions are preferably used as a second step: the internal transcribed spacer (ITS), notably for the differentiation of the members of the *Mycobacterium avium* intracellulare complex [13], and the *rpoB*, for the rapid growers [14]. Less commonly investigated targets include *recA* [15], *sodA* [16] and *gyrB* [17]. The analysis of a combination of sequences from several genes has been suggested to increase the discriminatory power [18].

The identification based on genetic sequencing relies on the use of programs able to compare the query sequence with the ones available in a nucleotide database. The most popular such program, BLAST (Basic Local Alignment Research Tool) [19], which is based on multisequence alignment, is capable, in few seconds, to find the more similar sequences among the ones available in the database.

The aim of this standard operating procedure is to propose an answer to the FAQ (frequently asked question) “I got a good quality 16S rDNA electropherogram from my *Mycobacterium* strain, how can I be confident that the quality of my identification is good, too?” It reflects the opinion of the author about the different steps that need to be taken into account when dealing with identification of mycobacteria using sequence data.

## The databases

The features of the database used play a central role. The available genetic databases can be grossly allotted to two major types: the controlled versus uncontrolled databases. Usually the libraries of the controlled databases, either commercial or free-access, include only validated sequences derived from reference strains. The best known commercial database can be accessed, by subscribers, through the MicroSeq ID Analysis software and is intended for the interpretation of the sequences obtained with the MicroSeq Bacterial Identification kits (Applied Biosystems) [20]. RIDOM (http://rdna2.ridom.de/) is the most popular free-access controlled database [21], well known is also EzTaxon [22] (http://147.47.212.35:8080).

Among uncontrolled databases, the partners of the international nucleotide sequence database collaboration (INSDC, http://www.insdc.org), comprising DDBJ (http://www.ddbj.nig.ac.jp/), EMBL (http://www.ebi.ac.uk/embl/) and GenBank, (http://www.ncbi.nlm.nih.gov/Genbank/) are well known worldwide.

Unfortunately, the qualitative standard of existing databases is nowadays inadequate. Controlled databases include sequences of excellent quality furthermore characterized by correct assignments at the species level; however, their inveterate lack of updates, along with the exclusion of any of the sequences presented by strains other than reference, heavily limit their usefulness. MicroSeq includes at present only 82 *Mycobacterium* species, while the number of officially recognized ones is now close to 150. In RIDOM, which has no more been updated since 2003 because of lack of funding, only 93 are present.

Differently from controlled databases, INSDC partners accept the external submission of any nucleic acid sequence of at least 50 bp. The advantages of this policy are the real-time updating of the database and the inclusion of sequences derived from thousands of organisms in adjunct to those of reference strains. The quality of sequences is sometimes unsatisfactory because of the presence of frequent ambiguities and because of their limited length. Even more disturbing is the presence of sequences arbitrarily labeled, at times with clearly erroneous species attribution. Furthermore, the frequent presence of multiple overlapping sequences clutters the database without any utility for the user.

Both lack of submitters’ updating and presence of incorrect entries may lead to major identification errors. The experience of the microbiologist may help to avoid these errors but remains powerless when an incomplete database is used. Although not optimal, INSDC partners represent the best tool available at present for the sequence-based identification of mycobacteria.

## From the sequence to species identification

Following the BLAST of the query sequence, two scenarios are possible depending on the presence or absence of one or more identical sequences in the database.

Contrary to common opinion, the label of a sequence 100% identical to the query cannot be assumed outright as the correct identification of the test strain. The presence of misassigned sequences in uncontrolled databases is well known [23]; in adjunct to gross errors (presumably due to sequence exchange at the moment of the submission), there are many sequences originating from very roughly characterized strains. Only in cases of complete identity to a sequence obtained from a reference strain, or better from a type strain, the identification can be relied upon, as the incidence of misidentifications among reference strains is quite low, albeit not zero [24,25].

A good number of sequences in INSDC partners are only labeled as belonging to the *Mycobacterium* genus; although useless for identification purposes, such entries still provide important information: some strains possibly identical to the query have been isolated by others who, in the absence of evidence, judiciously decided not to classify them at the species level.

An unresolved general taxonomic problem is the definition of the minimal sequence diversity exceeding the variability within a species and being sufficient to claim diversity of species; regarding mycobacteria, very poor consensus has been achieved using such cut-off levels for the 16S rRNA. The application of strict mathematical limits to biological entities is always questionable, all the more in the case of *Mycobacterium* taxonomy where almost 50% (no. 70) of the species officially recognized as distinct are characterized by genetic similarities >99% with one or more species of the genus; among them, several clusters of species even share identical 16S rDNA sequences (Table 1).

**Table 1:** *Mycobacterium* species sharing identical 16S rDNA sequences.

**Species**	**16S rDNA similarity %**	
	**first 500 bp**	**whole gene**
*M. abscessus*, *M. bolletii*, *M. chelonae*, *M. massiliense*	100	100
*M. abscessus*, *M. chelonae*	100	99.7
*M. africanum*, *M. bovis*, *M. bovis BCG*, *M. caprae*, *M. microti*, *M. pinnipedii*, *M. tuberculosis*	100	100
*M. alvei*, *M. setense*	100	99.1
*“M. barrassiae”*, *M. moriokaense*	100	99.6
*M. bolletii*, *M. chelonae*	100	99.7
*M. chelonae*, *M. massiliense*	100	99.7
*M. conceptionense*, *M. houstonense*	100	99.7
*M. conceptionense*, *M. senegalense*	100	100
*M. farcinogenes*, *M. fortuitum*, *M. houstonense*, *M. senegalense*	100	100
*M. gastri*, *M. kansasii*	100	100
*M. houstonense*,* M. senegalense*	100	100
*M. marinum*, *M. ulcerans*	100	99.8
*M. mucogenicum*, *M. phocaicum*	100	100
*M. murale*, *M. tokaiense*	100	100
*M. paraseoulense*, *M. seoulense*	100	100
*M. peregrinum*, *M. septicum*	100	99.7
*M. vaccae*, *M. vanbaalenii*	100	99.3

The impossibility of using a cut-off further complicates the identification of mycobacteria for which the BLAST does not find any 100% identical entries. The passive acceptance of the identification corresponding to the entry with the highest similarity may be responsible for misidentification in a substantial number of cases. The reliability of an identification based on a similarity ranging from ≥99 to <100%, is strongly dependent, besides the value of such percentage, on the characteristics of the database entry involved (type strain, clinical isolate, etc.). In case of similarity <99%, the probability of a correct identification becomes extremely low. The aforesaid percentages refer to the most common situation, in which a sequence spanning the first third of the 16S rDNA has been determined; the use of shorter sequences introduces a further variable affecting the confidence of the identification.

## Suggestions to the database user

**Figure 1:**
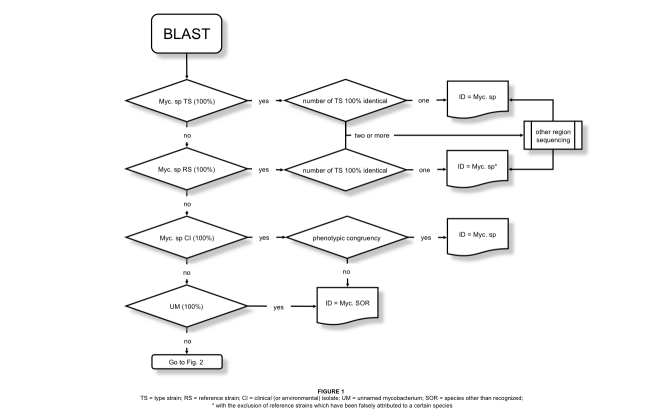
TS = type strain; RS = reference strain; CI = clinical (or environmental) isolate; UM = unnamed mycobacterium; SOR = species other than recognized; *with the exclusion of reference strains which have been falsely attributed to a certain species

**Figure 2:**
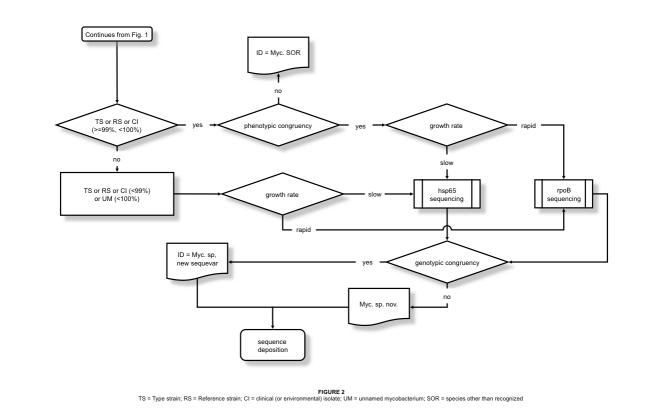
TS = type strain; RS = reference strain; CI = clinical (or environmental) isolate; UM = unnamed mycobacterium; OTRS = species other than recognized

The complexity of the path leading from a sequence to the identification (Figures 1 and 2) is evidently in contrast to the opinion of most microbiologists using genetic sequencing for the speciation of mycobacteria. The achievement of a good quality electropherogram is commonly considered the critical point of the whole process, while the identification itself is regarded as a mere formality with the first entry emerging from the database being automatically considered the optimal choice. Actually, the quality of the electropherograms is a matter of technical skill, the excellence of the identification is a matter of thought and insight, capabilities difficult to learn. The following short guidance aims to provide basic tips and to point out the most common traps.

- Every sequence present in INSDC partners comes with relevant annotations which offer precious hints. The frequently reported source of the strain allows the recognition of the more reliable reference strains from clinical or environmental isolates. The name of the submitter is important too, as his reputability allows grading the trustworthiness of the entries. The year of submission may be helpful, as very old sequences can be affected by the outdated techniques used. In the presence of identical sequences with different species attribution, one of which belonging to a novel species, it is common to see that the other had been submitted years before the description of the new species.
- Among the entries more closely related to the query sequence, it is prudent to take at first into account, if present, the one derived from a reference strain even if more similar ones do exist. A careful check of mismatches often substantially reduces their number as some of them are either due to the presence of ambiguous nucleotides in the database sequence or to incorrect interpretation of the electropherogram. Should the mismatches be confirmed the best choice is probably to leave the strain unidentified as its unsatisfactory similarity with the closest reference strain makes even more daring its attribution to other species whose reference strains present lower similarity.
- In the species with double ribosomal operon the possible presence of minor polymorphism between the two copies, although very rare, should not be neglected [26].
- An identification obtained in comparison with a reference strain should be pursued whenever possible, as there is no guarantee that the entries related to non-reference strains are correctly labeled. Therefore, it is at times better to disregard the best matching entry and to consider one of the more closely related reference strains, although it is characterized by a lower similarity percentage.
- The presence of multiple entries assigned to the same species, among the most closely related, increases the confidence of the identification. In contrast, the presence of a single entry related to the species “A” among several related to the species “B” should warn against the reliability of the first one. The possibility exists, however, of the presence of multiple sequences presenting the same incorrect attribution with the first erroneous submission misleading the subsequent submitters.
- For many sequences of reference strains this important information is sometime missing in the database; in cases in which a bibliographic reference is provided a quick glance at the paper can clarify the point.
- The presence of reference strains erroneously labeled, as they have been named in the pre-genomic era, is likely. The lack of a close similarity between their sequence and the one of the type strain should be considered suspiciously.
- Many species names associated to database entries lack standing in the literature; the consultation of the list of officially recognized *Mycobacterium* species (http://www.bacterio.cict.fr/m/mycobacterium.html) can make clear this point. In case of identity with one such sequence, the query organism remains unidentified. Unculturable mycobacteria, whose description as novel species is not possible, represent the exception to the rule.
- In the output of BLAST search, the order of entries does not always reflect the similarity score; it is recommended to order them by clicking on the header of the Maximum identity column.
- The detection of isolates possibly belonging to a novel species, whose sequence is identical to one already deposited by others, should lead to contact the submitters and to combine the strains. The amount of the sample is a major value in taxonomic studies while sp. nov. descriptions based on a single isolate are not advisable [1].

## Suggestions to the database submitter

Obviously the best help to the users of uncontrolled databases is provided by the quality of the submissions. From this point of view it is extremely important that the authors:

- Carefully check their submissions.
- Provide information about the nature of the strains (type strain, reference strain, clinical isolate, etc.).
- Update the submissions, by correcting any possible mistake, by updating the species name if it has been changed during the publication process, by giving references to relevant publications.
- Avoid labeling a sequence with a species name in cases in which such belonging is not evidence based.
- Avoid submitting multiple entries when they are identical or when an identical sequence is already present in the database, but systematically submit every new sequence found.
- Suggest to the author, or to the database editorial staff, the correction of any error detected in sequences or strain data.
- Avoid  giving tentative names to new strains prior the sp. nov. description.

An important issue to have clear in mind is that the improvement of uncontrolled databases relies almost solely on the auto-control of the submissions.
